# From Crisis to Coordination: Challenges and Opportunities for Integrated Care posed by the COVID-19 Pandemic

**DOI:** 10.5334/ijic.5595

**Published:** 2020-08-12

**Authors:** K. Viktoria Stein, Nick Goodwin, Robin Miller

**Affiliations:** 1 Karl Landsteiner Institute for Health Promotion Research, Vienna, AT; 2 Central Coast Research Institute, Faculty of Health and Medicine, University of Newcastle and Central Coast Local Health District, AU; 3 Central Coast Local Health District, and IFIC, AU; 4 Centre for Health & Social Care Leadership, University of Birmingham, UK; 5 International Journal of Integrated Care, AU

**Keywords:** collaborative policies, integrated care, vulnerable populations, COVID-19, health inequalities

Much has already been written on the impact and consequences of the COVID-19 pandemic, the rival merits of different approaches to managing the crisis, and the unpreparedness of most countries to tackle such an outbreak. Scientists are still struggling to understand the exact workings, ways of transmission, and diversity of symptoms caused by COVID-19. While we wait for a vaccine and treatments, the enormous impact of the lockdown around the world is coming to light with force.

The impact of COVID-19 has thrown into sharp relief the problems that fragmented health and care systems face in adapting to crises that require an urgent and collaborative response. The disproportionate impact of the pandemic – for example on ethnic minority and indigenous populations; on older people living in residential aged care facilities; on those living in rural and remote communities; on the poorest; and on people with the most complex health and care needs – says much about our continued inability to coordinate care and support our vulnerable communities, and so expose them to disproportionate risk.

For health and care systems, the impact of COVID-19 will also be long-felt – not just in terms of the immediate and ongoing pressures to respond to those needing treatment and rehabilitation, and indeed the significant build-up of unmet demand (e.g. in elective treatment), but in the challenges set for a necessary transition in care systems to adapt to a so-called ‘new normal’ way of operating.

Our response is hampered by the lack of essential data and knowledge. Any thorough analysis of the policies and their consequences will not be possible for some time to come. What we can say with certainty is that COVID-19 has demonstrated the shortcomings of our health and social systems, shortcomings exacerbated by political inertia in addressing complex societal challenges through population-based approaches. These shortcomings include fragmentation between and within public systems, be it health, education, housing or social services, the inexistent capacity to look at systemwide datasets, analyse them and draw conclusions from them, and the lack of cross-sectoral communication and planning. As with all crises, the most vulnerable in our societies are paying the price.

Despite numerous, and often inspirational examples, of the impact of COVID-19 eliciting change towards a more community-led and integrated response [1], we point to three persistent and well-known challenges which now need addressing.

## Challenge 1: Responses to COVID-19 have largely NOT been integrated, leading to adverse outcomes

The lack of preparedness for a pandemic lead most countries to shut down all health and social services except those directly dealing with the clinical treatment of COVID-19. As a consequence, many people with on-going, chronic and complex conditions did not receive any services at all for a prolonged period of time. This will cause an increase in exacerbations and frailty due to unmanaged chronic conditions, lack of rehabilitation, postponed procedures and complex social issues, with unintended consequences probably far outweighing the impact of the virus itself. The fact that care homes and home-based care were often forgotten in the beginning of the crisis further adds to the emerging narrative that our health and social systems are heavily skewed towards hospital and emergency services, and vulnerable populations along with the professionals caring for them have neither voice nor resources in this system. In some countries, decision making was further hampered by a lack of data gathering and data access for researchers and policy makers.

In order to future-proof our systems, we need to ensure that health and social services are maintained in times of crises. This does not call for more hospital beds, but on the contrary, an investment in strong and integrated primary health and social services, which follow a population-based approach, and support people at home and in their communities. The tragic stories of people dying alone because no one was allowed to visit them should be enough to ensure that this is never repeated, and we make sure people can die with dignity in the place of their choosing.

Crucially, COVID-19 must not be used as an argument to build more hospitals. An infectious disease pandemic is an exception, not the rule. What we need are better plans to tackle future outbreaks more effectively – for example, by anticipating the need for field hospitals, recognising the value and contribution of the local community, providing help and support for families, better coordination and communication between jurisdictions and governance levels. This means having a clear idea of the resources available in the health and social systems, having a common strategy of how to respond in a crisis, and having the data to support the decision-making process.

## Challenge 2: Responses continue to demonstrate inequalities in care and outcomes to vulnerable populations

The indirect impact of the months-long shutdown is likely to cause more harm, morbidity and mortality than the pandemic itself. The social determinants of health, especially unemployment, economic downturn, insecurity, and housing problems, will disproportionately affect the lower income tiers of society. This in turn will further widen inequalities, which amplified by the austerity policies following the financial crisis of 2008–9. Home schooling and distance learning disproportionately puts children and young people from low resource households at a disadvantage; home office becomes difficult when child care is unavailable and unaffordable; and the essential jobs defined during the crisis are also among the least paid, with precarious working conditions and high risk of infection.

COVID-19 has cruelly exposed the fact that those people who would most benefit from a coordinated response to their needs are the least likely to receive it. This is now likely to get worse, not better, without concerted action. COVID-19 should thus be seen as a wake-up call to radically change the policies on health and wellbeing. The WHO definition of health as “a state of complete physical, mental and social wellbeing” must be adopted as the guiding principle and ensure that population health is understood as the responsibility of everyone [2]. It has long been acknowledged that 90% of our health and wellbeing are influenced by factors other than access to clinical services [3] and it is time we acted on that.

## Challenge 3: The need to build evidence for an integrated and coordinated response, and to inform and drive policy and practice

This pandemic further underlines the need for more evidence-informed policy making and interdisciplinary decision making. Emergency doctors, virologists, consultants and mathematicians have important expertise, but we also need to hear from those who understand a people-centred approach, and can take into account the wider impact lockdowns and reduced public services will have on society. Public health experts, allied health professionals, and social workers among others should form an integral part of any advisory committee to formulate the way forward, as should civil society representatives. Research and evaluation need to be built-in requirements for any policy implementation, with transparent communication of results and lessons learned. We need to understand health and the management of crises as a continuous, emergent issue, with many unknowns, which require flexible and innovative approaches. In order to be able to learn from the crisis and better prepare for future outbreaks, we need to ask the right questions, invest in sound research and not sacrifice research principles due to the urgency and pressure of the crisis. This implies a research agenda that must go beyond the clinical and epidemiological to explore policy reforms and system responses where humanities and social sciences are prioritised.

## Conclusion

COVID-19 has accentuated the stark reality that, despite the efforts of the past 20 years, there remains a continued failure to embrace integrated care systems. It has also demonstrated how quickly systems, organisations and individuals can change, if they must. The uptake in telehealth, eHealth and other technological support systems is unprecedented, even though only a couple of months ago this was unthinkable on a broad scale in many countries. The swiftness of multi-disciplinary teams coming together to find flexible solutions to the day-to-day challenges during lockdown has shown how we can achieve effective communication and collaboration. The resilience and innovativeness of communities to help themselves has once again brought to light the importance of social networks and intact neighbourhoods. Care workers, often seen to have lower status than clinical professionals, have been lauded in their willingness to go far beyond their call of duty to ensure services are still delivered to those most in need.

Now is the time to make sure the challenges, sacrifices and innovations observed during the pandemic have not been in vain. We need to use this as an opportunity to change our systems for the better. COVID-19 is an example of what happens when systems are ill prepared, the workforce is overtaxed, and politicians are seemingly more concerned with their reputation than the health of the population. Instead of applauding our care workers, we need to pay them better and prepare them for a more integrated, coordinated and multi-disciplinary workspace in the community. Instead of pushing our vulnerable neighbours to the margins, we need to bring them into the centre of our communities and build a network of support around them. Instead of making short-term, politically motivated decisions, we need to ensure evidence-based and long-term strategies are in place, which define equitable health and wellbeing as outcomes for public service delivery.

COVID-19 has pushed our societies to the limit, but it has also shown us a way forward. As an international community of research and practice in integrated care, we must make sure not to waste this opportunity and be part of the change.
